# Relationship between lead concentration in maternal and umbilical cord blood and some neonatal outcomes in primiparous opium-dependent mothers in Zahedan, Southeast of Iran in 2022

**DOI:** 10.1186/s12884-023-06068-5

**Published:** 2023-10-25

**Authors:** Samira Khayat, Hamed Fanaei

**Affiliations:** Pregnancy Health Research Center, Zahedan University of Medical Sciences, Zahedan, Iran

**Keywords:** Opium, Lead, Pregnancy outcome, Umbilical cord, Blood, Infant, Neonate

## Abstract

**Background:**

There are limited studies on maternal and umbilical cord blood lead levels and their relationship with each other and with pregnancy outcomes in women addicted to opium. The present study was conducted with the aim of investigating the relationship between lead concentrations in maternal blood and umbilical cord blood with some neonatal outcomes in primiparous opium-dependent mothers.

**Method:**

The present study is a cross-sectional and analytical research. A total of 35 mothers addicted to opium and 35 women without addiction from Zahedan city of Iran participated in this study in 2022. Convenience sampling was done, lead levels were measured and pregnancy outcomes checked by a checklist to collect information. Data analysis was done by independent t-tests, Pearson’s correlation coefficient, Point-Biserial Correlation Coefficient, multivariate linear regression and multivariate logistic regression using SPSS version 26 software.

**Results:**

There was a significant difference in maternal blood lead levels (24.97 ± 8.45 in addicted mothers and 7.5 ± 4.25 in healthy mothers) and in umbilical cord blood lead concentrations (18.68 ± 6.95 in the addicted group and 5.7 ± 2.84 in the healthy group) between the two groups (P < 0.0001 and P < 0.0001, respectively).The lead concentration of all the samples in the addicted group was higher than the high-risk levels. Birth weight, Apgar score, admission to NICU and need for resuscitation were significantly different between the two groups (P < 0.0001, P < 0.0001, p = 0.03 and p = 0.04, respectively). Based on the results of multivariate linear regression test, only addiction can reversely predict the birth weight (Beta= -0.74, P < 0.0001), 1-minute Apgar score (Beta = -0.47, P = 0.004) and 5th minute (Beta= -0.62, P = 0.001). According to multivariate logistic regression test, OR of none of the predictor variables was significant with the outcomes. Maternal and umbilical cord blood lead concentrations were not predictors of pregnancy outcomes.

**Conclusion:**

The use of opium during pregnancy leads to an increase in the level of lead in maternal blood and umbilical cord blood. Addiction increased adverse pregnancy outcomes but lead concentration did not predict pregnancy complications. It is recommended that the addiction treatment program be included in pre-pregnancy and pregnancy care plans.

## Background

Opium is a source of lead pollution in Iran. Lead is a heavy metal, and considering the frequency of opium consumption, illegal drug dealers mix lead with opium to make more profit [1]. Evidence indicates an epidemic of lead poisoning in opium addicts in Iran [2, 3]. Pregnant women, infants and children are among the groups vulnerable to lead [4].

Statistics from UN Office on Drugs and Crime show that in 2020 61,000,000 people, namely 1.2% of the world’s population, used opioid drugs [5]. In Iran, according to the results of Noor Bala et al. study (2020), there were 2,340,000 addicts in the country, of which 4.6% used opioids and 7.6% of them lived in Sistan and Baluchistan Province [6]. Among those who abuse medicinal opioids, the proportion of women is significantly higher (47%) than for other drugs. In addition, 15% of opioid users are women [5]. There are no accurate statistics on the prevalence of addiction among Iranian women. In the study of Ramzanzadeh et al. (2007) in Tehran, the prevalence of drug use in pregnant women was 1.4% [7], and it was 0.69% in a study from Birjand [8]. According to a research conducted in the city of Fasa and the declaration of Iran’s Anti-Narcotics Organization, opium is the most common drug used by women in our country [9], which is unfortunately contaminated with lead due to profit seeking by traffickers [1] and can expose pregnancy to lead.

High amounts of lead have negative effects for pregnancy. Lead has a negative impact on the brain and nervous development of the fetus. It reduces fetal growth and increases the risk of miscarriage, premature birth and stillbirth as well as reducing the IQ of children [10] Lead is transferred from placenta to the fetus from the twelfth week of pregnancy [11], and high level of lead in the blood of a pregnant mother increases its level in fetal blood and causes complications on CNS during the fetal and neonatal periods [11, 12].

Studies show the contradiction between maternal and fetal plasma lead levels. In some studies, a positive and significant relationship has been reported between maternal and fetal lead levels [4, 13]. However, other studies have the opposite opinion. Hu et al. (2006) stated in their study that maternal blood lead levels are not a good indicator of fetal lead levels. Approximately 99% of lead in the blood is bound to red blood cells and is not free to pass through the placenta, and only 1% is plasma lead that affects the fetus, so maternal lead levels are an unreliable indicator of fetal blood lead levels [14].

Studies regarding the relationship between umbilical cord blood lead levels and pregnancy outcomes show that with increasing lead levels, hemoglobin concentration, gestational age at delivery, birth weight, height and head circumference of the baby are decreased, while the history of abortion, cesarean section (CS), premature rupture of membranes ( PPROM) and preterm delivery are increased [11, 15]. Also, a high level of lead in maternal serum is associated with an increase in abortion, stillbirth, malformation, infant death, and decreased brain development [4].

Pregnant mothers and fetuses are vulnerable to lead poisoning and there are contradictions regarding the relationship between lead levels in plasma of pregnant women and umbilical cord blood. There is insufficient information on the relationship between lead and pregnancy outcomes in women addicted to opium. Therefore, this study was conducted with the aim of determining the relationship between lead concentrations in maternal blood and umbilical cord blood and some neonatal outcomes in opium dependent mothers.

## Method

### Study design and participants

The current research is a cross-sectional study of analytical type that was conducted in maternity ward of Ali Ibn Abi Talib hospital in Zahedan city, Iran, in 2022. This study has been approved by ethics committee of Zahedan University of Medical Sciences (Ethical code: IR.ZAUMS.REC.1398.271).

The inclusion criteria were as follows: daily use of opium for at least one year, oral opium consumption, first pregnancy, physical and mental health, absence of heart disease, diabetes, kidney failure, high blood pressure, gestational age 37–42 weeks and living in Zahedan city. The following were exclusion criteria from the study: underweight, overweight and obesity at the beginning of pregnancy, maternal age < 20 and > 35 years, multiple pregnancy, pre-term and post-term pregnancy, use of other drugs, alcohol, cigarettes, hookah and methadone and exposure to second hand smoke.

### Sample size and sampling

A total of 70 pregnant women from Zahedan (35 without drug addiction and 35 addicted) participated in this research. To determine the sample size, Cohen’s table was used for the two-domain test; ά = 0.05, the power of test was 80% and the effect size was 0.7, the number of samples was estimated 33 people, and for more certainty, the number of samples in each group was 35 people. Sampling was done in the form of convenience sampling method. The samples were selected from among the mothers in maternity ward of Ali Ibn Abi Talib Hospital in Zahedan city. The samples of the addicted group were selected from those who had opium addiction and the control group participants were not opium addicts.

### Instruments

Demographic information form, atomic absorption spectrometry (AAS) method for lead measurement, digital scale, Apgar score table, observation of amniotic fluid color, and check list of pregnancy outcomes were used to collect data. To evaluate the lead levels, 1.5 ml maternal venous blood and 1.5 ml umbilical cord blood were collected from the studied groups in sterile heparin tubes. The lead level of the samples was measured by AAS method using GBC Avanta device with µg/ml accuracy. Weight was measured with a digital scale with an accuracy of 10 g, and the Apgar score was calculated based on pulse rate, breathing status, skin color, response to catheter insertion into the nose, and muscle tone. The observation of meconium with any concentration in the amniotic fluid during the delivery process was considered to be meconium amniotic fluid. A checklist including anomaly, hospitalization in NICU after birth, respiratory problems and need for resuscitation was used to investigate the outcomes of newborns.

### Data collection procedures

The researcher introduced herself and the objectives of research to the samples and then checked inclusion and exclusion criteria by asking the individual. Informed consent was obtained from the study participants. It was emphasized that the results would remain completely confidential, no information would be published bearing the person’s name, and their response would not affect the treatment measures. All methods were carried out in accordance with relevant guidelines and regulations.

Demographic questionnaires were completed; 1.5 ml of mother’s venous blood sample and 1.5 ml of umbilical cord blood were collected in a heparinized tube immediately after birth. The 1-minute and 5-minute Apgar scores of the babies were calculated by the researcher. The weight of the newborn was also measured by the researcher. During the delivery process, the researcher observed the amniotic fluid for the presence of meconium, and if the fluid was contaminated with any concentration, it was considered meconium.

After sampling, plasma was separated from the blood samples using a centrifuge at 3500 rpm for 10 min, and the plasma sample was kept in a freezer at -70 °C until the time of lead measurement. Then, the lead concentration was measured.

### Data analysis

The data was analyzed using descriptive and analytical statistical tests using SPSS version 26 software by considering the significance level of p < 0.05. Frequency, percentage, mean, standard deviation were used to describe demographic characteristics. For analytical purposes, the normal distribution of data was checked by Shapiro-Wilk test. Fisher’s exact test was used to compare the frequency of high-risk and low-risk lead levels in maternal blood and umbilical cord blood between the two groups. Lead levels in cord blood and maternal blood had a normal distribution in both groups. As a result, independent t-test was used to compare the mean concentration of lead in maternal blood and umbilical cord blood between the two groups. Pearson’s correlation coefficient was employed to determine the relationship between lead and other variables. Independent t-test, chi-square and Fisher’s exact test were utilized to compare pregnancy outcomes between the two groups. To determine the relationship between lead level and pregnancy outcomes, Pearson’s test was used for quantitative variables and Point-Biserial Correlation for nominal variables. Multivariate linear regression (in quantitative variables) as well as multivariate logistic regression (in binary variables) tests were used to simultaneously examine the effect of addiction and lead levels on pregnancy outcomes.

## Results

### Socio‑demographic characteristics and lead concentration in maternal and umbilical cord blood

A total of 70 pregnant women from Zahedan participated in this study. The average duration of opium use in the group of female addicts was 2.47 ± 2.82 years, and the amount of daily consumption was 885.71 ± 162.73 mg. Analysis results showed that the two groups did not differ significantly in terms of demographic characteristics (Table 1).

**Table 1:** Comparison of Socio‑demographic characteristics and lead concentrations in maternal blood and umbilical cord blood between the addicted mothers and the control group

Groups\ Variables		Control	Addicted	P value
		Mean ± SD	Mean ± SD	
Age (years)		21.28 ± 3.85	23.31 ± 4.91	0.06 ^a^
Body mass index (kg/m^2^)		22.22 ± 4.06	23.37 ± 3.16	0.14 ^a^
		Number (Percentage)	Number (Percentage)	
Education	Preparatory	15 (42.9)	22 (62.9)	0.09 ^b^
	Secondary	20 (57.1)	13 (37.1)	
Employment	Employed	31 (88.6)	33 (94.3)	0.67 ^c^
	Unemployed	4 (11.4)	2 (5.7)	
Maternal blood lead (µg/dL)	5>	11 (31.4)	0	< 0.0001 ^c^
	5≤	24 (68.6)	35 (100)	
Umbilical cord blood lead (µg/dL)	5>	15 (42.9)	0	< 0.0001 ^c^
	5≤	20 (57.1)	35 (100)	

a: Independent t-test, b: Chi-Square, c: Fisher’s Exact Test

According to Table 1, Fisher’s exact test showed that there was a significant difference in frequency distribution of high-risk and low-risk levels of lead in maternal and umbilical cord blood between opium addicted and healthy mothers (P < 0.0001 and P < 0.0001, respectively). The level of lead in mother’s blood and umbilical cord blood in all the samples of the addict group was at the high-risk level.

### Correlation of lead levels in maternal blood and umbilical cord blood

Based on Table 2, the results of independent t-test showed that there was a significant difference in maternal and umbilical cord blood lead levels between the group of addicted and healthy mothers (P < 0.0001 and P < 0.0001, respectively). Pearson’s test showed a significant relationship between maternal and umbilical cord lead levels in both groups (P < 0.0001).

**Table 2:** Lead level in mother’s blood and umbilical cord blood and their relationship with each other in the group of mothers addicted to opium and the control group

Groups\ Variables	Control	Addicted	P value
	Mean ± SD	Mean ± SD	
Maternal blood lead (µg/dL)	7.5 ± 4.25	24.97 ± 8.45	< 0.0001 ^a^
Umbilical cord blood lead (µg/dL)	5.7 ± 2.84	18.68 ± 6.95	< 0.0001 ^a^
P value	r = 0.79 P = < 0.0001^b^	r = 0.74 P < 0.0001^b^	

a: Independent t-test, b: Pearson Correlation

### Comparison of neonatal outcomes

Pregnancy outcomes were compared between the study groups and the results showed that birth weight, Apgar score, hospitalization of the baby in NICU and the need for resuscitation were significantly different in the two groups. However, there was no significant difference between the two groups in respiratory problems, meconium, and neonatal anomaly (Table 3).

**Table 3:** Comparison of neonatal outcomes between the study groups

Groups\ Adverse pregnancy outcomes		Control	Addicted	P value
		Mean ± SD	Mean ± SD	
Birth weight (gr)		3106 ± 424.41	2560 ± 342.63	< 0.0001 ^a^
1-minute Apgar score		8.25 ± 0.81	6.77 ± 1.11	< 0.0001 ^a^
5-minute Apgar score		8.97 ± 0.85	7.82 ± 0.92	< 0.0001 ^a^
		Number (Percentage)	Number (Percentage)	
Meconium	Yes	3 (8.6)	7 (20)	0.17 ^b^
	No	32 (91.4)	28 (80)	
Hospitalization in NICU	Yes	3 (8.6)	10 (28.6)	0.03 ^b^
	No	32(91.4)	25 (71.4)	
Respiratory problems	Yes	3 (8.6)	8 (22.9)	0.1 ^b^
	No	32 (91.4)	27 (77.1)	
Congenital anomalies	Yes	0 (0)	1 (2.9)	1 ^c^
	No	35 (100)	34 (97.1)	
Neonatal resuscitation	Yes	2 (5.7)	8 (22.9)	0.04 ^b^
	No	33 (94.3)	27 (77.1)	

a: T-test b: Chi-Square c: Fisher’s Exact Test.

### Correlation of lead levels in maternal blood and umbilical cord blood with pregnancy outcomes

Pearson’s correlation coefficient showed that there was a significant and negative relationship between maternal blood lead levels with birth weight and Apgar score. Also, there was a significant and negative relationship between cord blood lead levels with birth weight and Apgar score. The results of the relationship between lead concentrations and qualitative pregnancy outcomes showed that there was a significant relationship only between mother’s blood lead levels and NICU admission (Table 4).

**Table 4:** Correlation of lead levels in maternal blood and umbilical cord blood with pregnancy outcomes

Lead concentration\ Adverse pregnancy outcomes	Lead concentration in maternal blood		Lead concentration in umbilical cord blood	
	r	P value	r	P value
Birth weight (gr)	− 0.38	0.001	-0.39	0.001^a^
1-minute Apgar score	-0.56	< 0.001	-0.48	< 0.001 ^a^
5-minute Apgar score	-0.38	0.001	-0.43	< 0.001 ^a^
Meconium	-0.13	0.25	-0.1	0.4^b^
Hospitalization in NICU	-0.29	0.01	-0.17	0.14 ^b^
Respiratory problems	-0.16	0.18	-0.05	0.63 ^b^
Congenital anomalies	0.003	0.98	0.06	0.61 ^b^
Neonatal resuscitation	-0.2	0.09	-0.09	0.4 ^b^

a: Pearson Correlation, b: Point-Biserial Correlation

**Table 5:** The results of univariate and multivariate linear regression tests in determining the relationship between addiction, lead concentration in mother’s blood and umbilical cord blood with outcomes of weight and 1-minute and 5 min Apgar scores

**Birth weight (gr)**											
**Univariate regression**							**Multivariate regression**				
Variables		B	Beta	95% CI		P value	B	Beta	95% CI		P value
				Lower Bound	Upper Bound				Lower Bound	Upper Bound	
(Constant)							3040.61		2869.94	3211.28	< 0.0001
Addiction	Yes	-541.	-0.58	-725.12	-357.6	< 0.0001	− 695.02	− 0.74	-1000.66	-381.39	< 0.0001
	No	reference									
Maternal blood lead (µg/dL)		-16.43	-0.38	-25.95	-6.91	0.001	10.02	0.23	-11.15	31.19	0.34
Umbilical cord blood lead (µg/dL)		-22.15	− 0.39	-34.58	-9.71	< 0.0001	-1.6	-0.02	-28.3	25.04	0.9
**1-minute Apgar score**											
**Univariate regression**							**Multivariate regression**				
Variables		B	Beta	95% CI		P value	B	Beta	95% CI		P value
				Lower Bound	Upper Bound				Lower Bound	Upper Bound	
(Constant)							8.39		7.96	8.81	< 0.0001
Addiction	Yes	-1.48	-0.23	-1.95	-1.02	< 0.0001	-1.16	-0.47	-1.94	-0.37	0.004
	No	reference									
Maternal blood lead (µg/dL)		-0.06	− 0.56	-0.08	-0.04	< 0.0001	-0.04	− 0.44	-0.1	0.004	0.06
Umbilical cord blood lead (µg/dL)		-0.07	-0.48	-0.1	− 0.04	< 0.0001	0.04	0.28	-0.02	0.1	0.21
**5-minute Apgar score**											
**Univariate regression**							**Multivariate regression**				
Variables		B	Beta	95% CI		P value	B	Beta	95% CI		P value
				Lower Bound	Upper Bound				Lower Bound	Upper Bound	
(Constant)							8.9		8.51	9.3	< 0.0001
Addiction	Yes	-1.14	-0.54	-1.56	-0.71	< 0.0001	-1.3	-0.62	-2.02	-0.57	0.001
	No	reference									
Maternal blood lead (µg/dL)		-0.03	-0.38	-0.05	-0.01	0.001	0.03	0.36	-0.01	0.08	0.16
Umbilical cord blood lead (µg/dL)		-0.05	-0.43	-0.08	-0.02	< 0.0001	-0.03	-0.27	-0.09	0.02	0.26

B: Unstandardized Coefficients, Beta: Standardized Coefficients, CI: Confidence Interval

**Table 6:** The results of univariate and multivariate logistic regression tests in determining the relationship between addiction, lead concentrations in mother’s blood and umbilical cord blood with outcomes of meconium, hospitalization in NICU, respiratory problems, congenital anomalies and neonatal resuscitation

**Meconium**											
Variables		**Univariate logistic regression**					**Multivariate logistic regression**				
		B	OR	95% CI		P value	B	OR	95% CI		P value
				Lower Bound	Upper Bound				Lower Bound	Upper Bound	
(Constant)							-2.25	0.1			00.1
Addiction	Yes	0.98	2.66	0.62	11.3	0.18	1.22	3.41	0.43	27.11	0.24
	No	reference									
Maternal blood lead (µg/dL)		0.03	1.03	0.97	1.09	0.25					
Umbilical cord blood lead (µg/dL)		-2.21	0.1	0.95	1.11	0.001	-0.01	0.98	0.87	1.1	0.74
**Hospitalization in NICU**											
Variables		**Univariate logistic regression**					**Multivariate logistic regression**				
		B	OR	95% CI		P value	B	OR	95% CI		P value
				Lower Bound	Upper Bound				Lower Bound	Upper Bound	
(Constant)							-2.64	0.07			< 0.001
Addiction	Yes	1.45	4.26	1.06	17.16	0.04	0.8	2.22	0.26	18.86	0.46
	No	reference									
Maternal blood lead (µg/dL)		0.06	1.06	1.03	1.1	0.02	0.16	1.17	0.99	1.39	0.06
Umbilical cord blood lead (µg/dL)		0.05	0.11	0.98	1.12	0.14	-0.17	0.83	0.68	1.03	0.09
**Respiratory problems**											
Variables		**Univariate logistic regression**					**Multivariate logistic regression**				
		B	OR	95% CI		P value	B	OR	95% CI		P value
				Lower Bound	Upper Bound				Lower Bound	Upper Bound	
(Constant)							-2.38	0.09			0.001
Addiction	Yes	1.15	3.16	0.76	3.16	0.11	1.1	3.02	0.36	25.15	0.3
	No	reference									
Maternal blood lead (µg/dL)		0.03	0.09	0.98	1.03	0.18	0.003	1.003	0.91	1.09	0.95
Umbilical cord blood lead (µg/dL)		0.01	1.01	0.94	1.09	0.63					
**Congenital anomalies**											
Variables		**Univariate logistic regression**					**Multivariate logistic regression**				
		B	OR	95% CI		P value	B	OR	95% CI		P value
				Lower Bound	Upper Bound				Lower Bound	Upper Bound	
(Constant)											
Addiction	Yes	17.67	4751	0	1.1	0.99					
	No	reference									
Maternal blood lead (µg/dL)		-0.002	0.99	0.83	1.19	0.98					
Umbilical cord blood lead (µg/dL)		-0.08	0.92	0.66	1.28	0.62					
**Neonatal resuscitation**											
Variables		**Univariate logistic regression**					**Multivariate logistic regression**				
		B	OR	95% CI		P value	B	OR	95% CI		P value
				Lower Bound	Upper Bound				Lower Bound	Upper Bound	
(Constant)							-2.83	0.05			< 0.0001
Addiction	Yes	1.58	4.88	0.95	24.97	0.056	1.52	4.58	0.46	44.99	0.19
	No	reference									
Maternal blood lead (µg/dL)		0.05	1.05	0.99	1.15	0.1	0.004	1.004	0.91	1.09	0.93
Umbilical cord blood lead (µg/dL)		0.03	1.03	0.95	1.11	0.42					

B: unstandardized coefficient, OR: Odd Ratio, CI: Confidence Interval

Multivariate linear regression analysis was used to estimate the effect of addiction and lead concentration in maternal blood and umbilical cord blood on birth weight and Apgar score 1 and 5 min. First, univariate linear regression was performed, and the variables that had a significance level of < 0.2 were entered into the multivariate regression model. According to the results of Table 5, in all three outcomes of birth weight, 1-minute and 5 min Apgar scores, only the Beta coefficient of addiction was significant in the < 0.05 level (Beta= -0.74, Beta = -0.47 and Beta= -0.62, respectively). As a result, among the regression predictor variables, only addiction can reversely predict the birth weight, Apgar score 1st and 5th minute.

To estimate the effect of addiction and lead concentrations in maternal blood and umbilical cord blood on binary variables (meconium, NICU admission, respiratory problems, congenital anomalies and neonatal resuscitation), multivariate logistic regression analysis was used. First, univariate logistic regression was performed, and the variables that had a significance level of < 0.2 were entered into multivariate regression model. According to the results of Table 6, the OR of none of the predictor variables was significant with the outcomes.

## Discussion

The results of the present study showed that the blood lead level of mothers addicted to opium is significantly higher than that of healthy women and that the concentration of lead in mother’s blood in all addicted mothers is in the high-risk range. In line with the results of the present study, the research by Rezaei et al. (2019) showed that the level of lead in blood of mothers using drugs (opioid, methadone, heroin, methamphetamine) was significantly higher than that of healthy women [16]. Ghaemi et al.‘s (2017) study confirms that in opium addicts, the level of lead in the serum is significantly higher than that in healthy people [17]. However, the results of Hayatbakhsh Abbasi’s study did not find a significant difference in serum lead levels of opium addicts and healthy people [18], which may be due to the difference between the society and the place of research with the present study.

The review of studies shows that no study has investigated the concentration of lead in umbilical cord blood of newborns from addicted mothers. However, the results of the present study showed a significant increase in the level of lead in umbilical cord blood of opium-addicted pregnant mothers. In all the examined cord blood samples, the lead level was higher than 5 µg/dL. A survey of pregnant Iranian women shows that in 38.5% of them, the level of lead in umbilical cord blood exceeds the permissible limit of 5 µg/dL [19]. In the current study, the average concentration of lead in the umbilical cord blood of addicted people was 18.68 µg/dL, which is higher than the permissible level.

Lead easily passes through the skin and digestive system and is absorbed through breathing, and after entering the plasma, it passes through the blood-brain barrier and the placenta and deposited in soft tissues. The half-life of lead in blood is 25–37 days, in soft tissue 40 days, and in bone 25–40 years [20]. Approximately 90% of lead is stored in the bones for years [21] during pregnancy, and due to its release from the bone sources of the mother, the fetus is at risk [22]; therefore, the stored lead can endanger the fetus years after exposure of the mother [21].

It is possible that the addition of lead to opium may present a significant hazard for lead exposure in infants, even after prolonged periods of time. The study also suggests that there is a notable presence of lead in the blood of opium-addicted mothers and their umbilical cord blood.

The statistical analysis of the present study regarding the relationship between maternal blood and umbilical cord blood lead levels also shows that there is a significant relationship between these two levels. In line with the present research, the study of Nejad chehrazi et al. (2011) [13] and that of Mahdi et al. (2023) [19] have reported a strong relationship between the two. However, this relationship was not seen in Hu et al.‘s (2006) study [14]. Lead passes through the placenta easily and without any obstacles [23]. For this reason, there is a significant relationship between the level of lead in the mother’s blood and in the umbilical cord blood, so the fetus is at risk of exposure to lead.

Exposure to opium during pregnancy has a number of negative consequences. According to the results of the present study, there was a significant difference between the two groups in terms of birth weight, Apgar score, NICU admission, and neonatal resuscitation. However, there was no significant difference between the two groups in terms of meconium amniotic fluid, respiratory problems and anomalies. The study of Fanaei et al. (2020) showed that there is a significant difference between meconium excretion, respiratory problems, neonatal resuscitation, anomaly, Apgar score, birth weight and NICU admission in opium-addicted and healthy women [24]. In Rahi et al.‘s study, Apgar score and birth weight were lower in addicted women than in healthy women [25].

Derakhshan et al. (2014) in Rafsanjan investigated the pregnancy outcomes of opium-addicted women. In their study, opium-addicted and healthy groups had a significant difference in terms of birth weight, but no significant difference was reported in terms of Apgar score and anomalies [26]. Intervening factors can account for the difference in these results. Addicts and healthy people are different in terms of quality of life and income. Addicted women receive less prenatal care and experience more sexually-transmitted diseases. Women’s lifestyle, including the use of other addictive tobacco products, is also a factor in creating these differences [26].

Lead is among the factors that can be related to the consequences of opium. Intrauterine life is the most vulnerable period of lead poisoning for the developing fetus [25].

The findings of the current study suggest that there is a correlation between the levels of lead in maternal blood and umbilical cord blood, and pregnancy outcomes such as birth weight, Apgar score, and NICU admission. However, when evaluating the combined effect of addiction and lead on pregnancy outcomes, it was observed that the increase in lead levels among addicted women did not lead to an increase in neonatal complications. Instead, it was found that addiction itself predicted a decrease in birth weight and lower 1-minute and 5-minute Apgar scores.

This indicates that while lead exposure may impact pregnancy outcomes, the relationship between addiction and these outcomes seems to be independent of lead levels. These findings emphasize the need for further research to better understand the complex interactions between addiction, lead exposure, and pregnancy outcomes.

A study in Shiraz among addicted and healthy women showed that there was no relationship between blood lead levels > 5 µg/dL and lower levels in terms of gestational age, anthropometric characteristics and Apgar score and that the only difference was in respiratory problems [16].

The results of meta-analyses by Sezavar et al. (2022) and Wang et al. (2020) show that there is a relationship between high lead levels and low birth weight [27, 28] and that levels > 0.3 µg/dL directly increase the possibility of low birth weight [27].

Chen et al.‘s (2006) study also found a blood lead level of 20 µg/dL to be associated with small for gestational age (SGA) [29]. Akbari-Nassji’s study (2017) in Abadan city showed that there is no relationship between cord blood lead concentrations and birth weight [30]. Golmohammadi et al.‘s (2007) study on women in lead-contaminated and non-contaminated cities of Iran showed that maternal blood lead levels are not related to birth weight [20]. Dalili et al. (2019) reported that there was no correlation between the lead levels in umbilical cord blood and mother’s blood with the weight, height and head circumference of the baby in Tehrani mothers [21]. A study in Nigeria also showed that there is no relationship between lead levels in mother’s blood and in umbilical cord blood with infant’s anthropometric indicators [22]. Studies show contradictions regarding the relationship between lead and pregnancy outcomes, and there is a need for further research in this field.

### Limitations

Due to the sensitivity of the subject of opium use, there is a possibility that the samples did not honestly report the use. The researcher tried to convince the samples by fully explaining the objectives of the study and observing the ethics in the research so that the information is completely confidential and does not affect the treatment process.

## Conclusion

Based on the findings of the current study, it can be concluded that the utilization of opium during pregnancy is associated with an elevation in lead levels in both maternal blood and umbilical cord blood. There exists a direct correlation between lead levels in the mother’s blood and umbilical cord blood. The outcomes of pregnancy differ between opium addicts and individuals who are not addicted, with birth weight, Apgar score, and respiratory issues being influenced by lead levels. The increased release of lead from the mother’s bones during pregnancy due to opium use exposes the fetus to both endogenous and exogenous lead, thereby increasing the likelihood of complications. However, it is important to note that addiction, rather than concentrations of lead in maternal blood or umbilical cord blood, serves as the predictive factor for neonatal complications. Consequently, it is advisable to incorporate addiction treatment programs into pre-pregnancy care initiatives as well as during pregnancy.

## Data Availability

The datasets used and/or analyzed during the current study are available from the corresponding author on reasonable request.

## Abbreviations

NICU: Neonatal Intensive Care Unit
UN: United Nations
IQ: Intelligence Quotient
CS: Cesarean Section
PROM: Premature Rupture Of Membranes
AAS: Atomic Absorption Spectrometry
